# The Effect of the Treatment with Heated Humidified High-Flow Nasal Cannula on Neonatal Respiratory Distress Syndrome in China: A Single-Center Experience

**DOI:** 10.1155/2017/3782401

**Published:** 2017-01-12

**Authors:** Ge Zheng, Xiao-qiu Huang, Hui-hui Zhao, Guo-Xing Jin, Bin Wang

**Affiliations:** ^1^Southern Medical University, Guangzhou, Guangdong Province, China; ^2^Department of Paediatrics, Ruian People's Hospital, Wenzhou, Zhejiang, China; ^3^Department of Paediatrics, Zhujiang Hospital of Southern Medical University, Guangzhou, Guangdong Province, China

## Abstract

*Background*. Noninvasive respiratory support is considered the optimal method of providing assistance to preterm babies with breathing problems, including nasal continuous positive airway pressure (NCPAP) and humidified high flow nasal cannula (HHHFNC). The evidence of the efficacy and safety of HHHFNC used as the primary respiratory support for respiratory distress syndrome (RDS) is insufficient in low- and middle-income countries.* Objective*. To investigate the effect of heated humidified high flow nasal cannula on neonatal respiratory distress syndrome compared with nasal continuous positive airway pressure.* Methods*. An observational cross-sectional study was performed at a tertiary neonatal intensive care unit in suburban Wenzhou, China, in the period between January 2014 and December 2015.* Results*. A total of 128 infants were enrolled in the study: 65 in the HHHFNC group and 63 in the NCPAP group. The respiratory support with HHHFNC was similar to that with NCPAP with regard to the primary outcome. There is no significant difference between two groups in secondary outcomes. Comparing with NCPAP group, the incidence of nasal damage was lower in HHHFNC group.* Conclusions*. HHHFNC is an effective and well-tolerated strategy as the primary treatment of mild to moderate RDS in preterm infants older than 28 weeks of GA.

## 1. Introduction

Neonatal respiratory distress syndrome (RDS) is one of the most common morbidities in preterm infants and may be treated with noninvasive respiratory support such as nasal continuous positive airway pressure (NCPAP) [1]. However, the use of NCPAP may lead to different degrees of damage to the nose, such as nasal swelling, nasal septum necrosis, and other complications, some even requiring surgery [2].

Humidified high-flow nasal cannula (HHFNC) is getting popular as a modality of noninvasive respiratory support for preterm infants [3]. Some evidence from early studies confirms that the use of HHHFNC may be linked with reduced work of breathing, benefited from ventilation, and reduced the demand for intubation in infants with respiratory distress syndrome [4]. Despite its increasing popularity, only a few large randomized clinical trials (RCTs) have been carried out to assess the efficacy and safety of HHHFNC in newborn infants in the world. Most of the RCT were performed after extubation and in larger infants [5]. Thus, there is a need for more data on primary therapy for RDS, especially in middle-income countries. It can hardly be denied that China's medical resources and number of healthcare professionals are still insufficient and HHHFNC might be of great importance in middle-income countries. The objective of our study is to investigate the effect of heated humidified high-flow nasal cannula on neonatal respiratory distress syndrome (RDS) compared with nasal continuous positive airway pressure as the primary noninvasive respiratory support.

## 2. Methods

This is an observational cross-sectional study performed on 128 preterm infants who are categorized into two groups; the first group received NCPAP and the second group received HHHFNC. This study was performed in a level III neonatal intensive care unit (NICU) in suburban Wenzhou, Zhejiang Province, China, in the period between January 2014 and December 2015.

Infants were eligible for the study if they matched the following inclusion criteria: (1) GA of 28 weeks 0 days (28^+0^ weeks) to 34 weeks 6 days (34^+6^ weeks) and birth weight >1000 g; (2) mild to moderate RDS requiring noninvasive respiratory support, characterized by a fraction of inspired oxygen (FiO_2_) lower than 0.3 for target saturation of peripheral oxygen (SpO_2_) 88% to 93%; (3) parental consent obtained. Patients were ineligible if they presented with the following: (1) signs of serious, life-threatening malformations; (2) severe RDS requiring early intubation according to the American Academy of Paediatrics guidelines for neonatal resuscitation [6]; (3) severe intraventricular hemorrhage.

Provision of controlled early neonatal respiratory distress syndrome (CPAP) (T-piece) is now the main way of providing safe stabilization of preterm babies immediately after birth in our hospital. Babies with RDS should be given rescue surfactant early in the course of the disease. A suggested protocol would be to treat babies >26 weeks when FiO_2_ requirements >0.40 [7]. Infants who met prespecified criteria received surfactant via an INSURE (Intubation, Surfactant Administration, Extubation) technique. We enrolled preterm infants at the time of application of NCPAP or HHHFNC. The decision of putting the baby on NCPAP or HHHFNC was according to the attending neonatologist's decision; both modalities are used in our NICU for neonates requiring respiratory support.

The study was approved by the local medical research ethics committee and written informed consent of parents was obtained. HHHFNC was delivered by the Optiflow Junior (Fisher & Paykel Healthcare, New Zealand) or Vapotherm (Vapotherm, Exeter, USA). Nasal CPAP support was provided by the Arabella (Hamilton Medical, Inc., Bonaduz, Switzerland) utilizing pressures ranging from 3 to 8 cm H_2_O.

Infants assigned to the HHHFNC group received an initial gas flow of 6 to 8 liters per minute. The size of the nasal cannula was determined according to the manufacturers' instructions in order to maintain a leak at the nares. The maximum permissible gas flow was 8 liters per minute, as recommended by the manufacturer. In the infants assigned to NCPAP, the starting pressure was 4 to 6 cm of water, achieved with a ventilator. Treatment was delivered through short binasal prongs, with sizing determined according to the manufacturer's recommendations. The maximum permissible pressure was 8 cm of water. Changes in respiratory support were made in steps of 1 liter per minute (for high-flow therapy) or 1 cm of water (for NCPAP). All infants were evaluated at least once a day.

Weaning from noninvasive respiratory support was considered if there was clinical improvement and the infants were receiving a fraction of inspired oxygen of 0.3 or lower, whereas discontinuation of noninvasive support was considered in infants who were receiving a fraction of inspired oxygen of 0.3 or lower, with gas flow of 4 liters per minute (in the HHHFNC group) or pressure of 5 cm of water (in the NCPAP group); earlier cessation of support could be ordered at the discretion of the treating clinician.

The primary outcome is intervention failure within 7 days after noninvasive respiratory support defined as requiring endotracheal intubation and mechanical ventilation. Secondary outcomes include the incidence of bronchopulmonary dysplasia (BPD) (requirement for supplemental oxygen and/or respiratory support at 36 weeks' postmenstrual age (PMA) for infants born at less than 32 weeks' gestational age or at 28 days of age for infants born at 32 weeks' gestational age or later), pneumothorax, severe intraventricular hemorrhage (IVH, Papile's grade 3 or 4), retinopathy of prematurity (ROP), nasal trauma, time until full feeds (when full enteral feeding was achieved ≥120 mL/kg per day), late-onset sepsis, necrotizing enterocolitis (NEC), and length of stay.

The criteria for intubation were the following: (1) apnea despite 30 seconds of positive pressure ventilation; (2) FiO_2_ greater than 0.6 to maintain SpO_2_ more than 88%; (3) persistent marked/severe retractions; (4) cardiovascular collapse (heart rate less than 60 beats per minute or shock); (5) severe metabolic acidosis (arterial base deficit less than −10); (6) severe respiratory acidosis (arterial PCO_2_ more than 65 mmHg).

## 3. Statistical Analysis

The data are normally distributed continuous variables expressed as mean ± standard deviation, skewness distribution by median (interquartile range) representation. Normally distributed continuous variables between groups were compared using Student's *t*-test, skewed distribution by test using Wilcoxon Mann–Whitney *U* test. Dichotomous outcomes were compared by *χ*^2^ test or Fisher's exact test. Two-sided *P* values 0.05 were considered statistically significant, and no adjustments were made for multiple comparisons. All analyses were performed with the use of SPSS (version 19; IBM, Armonk, NY).

## 4. Results

A total of 128 infants were involved in the study between January 2014 and December 2015, 65 in the HHHFNC group and 63 in the NCPAP group. The baseline characteristics of the two groups were similar at the time of treatment (Table 1). The group of HHHFNC was similar to the NCPAP group with regard to the primary outcome (Table 2). Regarding the failure from the start of the study within 7 days of noninvasive respiratory support, in the group of HHHFNC of 65 patients, 13 cases failed (20%), while in the NCPAP group of 63 infants, 11 cases (17.5%) failed (95% CI of risk difference, 0.5% to 2.9%; *P* = 0.71) (Table 2). There was no significant difference in failure rates between the 2 groups at any of the gestational age (Table 2).

There were no significant differences between the 2 groups in most of the secondary respiratory outcomes except for nasal trauma rates (Tables 3 and 4). The HHHFNC and NCPAP groups were similar in overall duration of respiratory support (median [IQR], 5.6 [3.0 to 15.0] versus 5.1 [2.0 to 14.0] days; 95% CI of difference in medians, −0.32 to 1.33; *P* = 0.72), days of noninvasive respiratory support (median [IQR], 5.2 [3.0 to 13.0] versus 4.8 [2.0 to 13.0] days; 95% CI of difference in medians, −0.25 to 1.06; *P* = 0.31), days of oxygen supplementation (median [IQR], 0.4 [0.0 to 3.0] versus 0.3 [0.0 to 3.0]; 95% CI of difference in medians, −0.19 to 0.38; *P* = 0.26), need for surfactant (38.5% versus 42.9%; 95% CI of risk difference, 0.41 to 1.69; *P* = 0.61), duration of caffeine treatment (median [IQR], 3.2 [0.0 to 24.0] versus 2.0 [0.0 to 22.0] days; 95% CI of difference in medians, −0.23 to 2.51; *P* = 0.68), and the rate of air leaks (3.1% versus 1.6%; 95% CI of risk difference, 0.17 to 22.3; *P* = 0.58). Finally, we found no significant difference between the two groups in the rate of BPD (9.2% versus 9.5%; 95% CI of risk difference, 0.29 to 3.2; *P* = 0.96) (Table 3). Any acute adverse events besides air leaks and long-term complications of prematurity were strictly monitored after study entry. The 2 groups did not show significant difference for any of them (Table 3). The total number of deaths is two: 1 case in the HHHFNC group (28 weeks, died at 40 days with septic shock) and the other case in the NCPAP group (29 weeks at 16 days with NEC). The overall rate of sepsis was similar between the 2 groups. The combined outcome of “any adverse event” was not significantly different between the 2 groups. The rate of nasal trauma was significantly lower in the HHHFNC group than that in the NCPAP group (21.5% versus 42.9%; 95% CI of risk difference, 0.17 to 0.79; *P* = 0.01) (Table 4). Finally, no statistically significant differences were found in duration of hospitalization, full enteral feeding, weight, or exclusive breastfeeding at discharge (Table 4).

## 5. Discussion

In this study, we did not find significant differences in neonates older than 28 weeks of gestational age receiving noninvasive respiratory support with HHHFNC or NCPAP in the primary outcome: treatment failure within the first 7 days. In addition, we found no difference between two groups in respiratory support results, including oxygen supplementation time, diagnosing BPD, or hospital discharge for oxygen. Finally, we found that HHHFNC was associated with less nasal trauma than NCPAP.

Noninvasive respiratory support including NCPAP and HHHFNC [8] is considered the optimal method of providing assistance to preterm babies with breathing problems. HHHFNC has gained popularity all over the world in the recent years. The 2015 survey of UK [9] shows that the use of HHHFNC significantly increased in 2015 (87%) compared with 2012 (56%). There is insufficient evidence about the safety and efficacy of HHHFNC in low- and middle-income countries. Most studies investigated the heated humidified high-flow nasal cannula for the prevention of extubation failure in neonates [10–13]. The evidence of respiratory support of HHHNFC as primary mode after birth in preterm infants is rare [14–16]. Lavizzari et al. [14] conducted a large RCT on HHHFNC versus NCPAP in infants between 29 and 36 weeks' GA as primary therapy to mild to moderate RDS in preterm infants. In their study, HHHFNC showed efficacy and safety similar to those of NCPAP when applied as a primary approach to mild to moderate RDS in preterm infants older than 28 weeks' GA. Despite the different study design and lower percentages of infants less than 32 weeks and less than 1500 g in our study (*P* = 0.05, *P* = 0.126), in agreement with Lavizzari's RCT [14], we draw the same conclusion that HHHFNC is an effective and well-tolerated strategy that could be as effective as NCPAP as the primary treatment of mild to moderate RDS in preterm infants older than 28 weeks' GA. On the contrary, the HIPSTER study [17] showed that high-flow therapy resulted in a significantly higher rate of treatment failure than NCPAP when used as primary support for preterm infants with respiratory distress. However, there is significant difference between two studies in the baseline characteristics of the study population including the proportion of gestational age < 32 weeks: 51.2% in the HIPSTER study versus 32.8% in our study, respectively. This indicates that the more mature preterm infants may tolerate HHHFNC better as the primary support for preterm infants. But higher failure rates occurred in our study, because of the low rate of prenatal steroids and caffeine treatment. Another reason might be that the time of treatment failure of our study is within 7 days instead of 72 hours. The rate of neonatal resuscitation was also higher (~58%) in these relatively large infants in our study because the rate of prenatal steroids was relatively low (38.3%), which is the routine in middle-income countries. In agreement with the previous large RCTs on HHHFNC [14, 17], we did not find any difference in the rate of sepsis when compared with NCPAP.

The model of respiratory support of HHHFNC was studied as a main mode of respiratory support in the delivery room [18]. Heated, humidified high-flow nasal cannulas (HHHFNC) are small, thin, tapered binasal tubes that deliver oxygen or blended oxygen/air at gas flows of more than 1 L/min [5]. Although HHHFNC is a relatively simple device, an important drawback is the inability to be certain of the delivered airway distending pressure [19]. The primary safety concern with HHHFNC is the potential for high, unmeasured distending pressures. In an in vitro model, Sivieri et al. recorded pressures up to 20–30 cm H_2_O with flow rates >2 L/min [20]. The pressure generated in HHHFNC was not measured during this study. There are also concerns about the potential of infection in the use of HHHFNC [21]. Despite these drawbacks, we found a low rate of air leaks in the group of HHHFNC similar to previous studies (*P* = 0.58) [16, 22]. Similar to the study of De Klerk [23], we found that HHHFNC are smaller and lighter and typically utilize short, nonocclusive binasal prongs and require use of a heated water humidifier to prevent nasal trauma. But the rate of nasal trauma was relatively high in both groups in our study as compared to the current literature [10, 17], and this may be related to the limited number of paediatricians and nurses in China. China Health Statistics Almanac and World Health Statistics estimate that China had only 0.43 paediatricians for every 1000 children in 2012 and 2.05 nurses per 1000 population in 2013, well below the world average of 2.86 nurses [24]. The average doctor-to-nurse ratio in our hospital surveyed was 1 : 1.6, and the average bed-to-nurse ratio was 1 : 0.6. The average doctor-to-nurse and bed-to-nurse ratio in the NICU in our hospitals surveyed were significantly lower than the Ministry of Health standard, indicating a serious nursing shortage in the NICU. The shortages are exacerbated by a high turnover rate of staff caused by heavy workloads, deteriorating doctor-patient relationships, and increased work related stress. The study of Wilkinson et al. [5] shows that the popularity of HHHFNC seems to be due to other perceived advantages; for example, the cannulas are easier to apply than NCPAP prongs, may be more comfortable for infants, may be associated with less nasal trauma, and may enable easier access to babies' faces, thus allowing for greater opportunities for feeding and parental bonding.

Our study had some limitations. It was a monocentric rather than multicentric RCT. The mode of support assignment could not be blinded to the medical team. Using objective failure criteria and management protocols reduces the possibility of a bias that this might have caused. On the basis of data from our center, we estimated that treatment failure within 7 days would occur in 17.5% of infants assigned to receive NCPAP. We preestablish a noninferiority margin for high-flow treatment of 10 percentage points above the failure rate for NCPAP treatment. High-flow therapy would be considered noninferior to NCPAP if the difference in the risk of treatment failure and the upper limit of the two-sided 95% confidence interval were less than 10% and the lower limit of the 95% confidence interval was below zero. For the study to have 90% power, a sample of 760 infants would be required. Thus, it is possible that our study was underpowered. The sample size of our study population may not have been large enough to completely rule out a beneficial effect of either mode of nasal support (type II error). The safety conclusions from our study should also be taken with caution because of small sample size, as our study did not have sufficient statistical power to detect differences in relatively infrequent complications such as air leak, NEC, PDA, and IVH. The study was underpowered for superiority but equivalence was found. Although this study is limited by its relatively small size, the data presented here indicate that HHHFNC may represent a similarly well-tolerated and effective alternative respiratory support mode to NCPAP in the preterm infant population with mild to moderate RDS. Its potential advantages include its simplicity, improved tolerability with less injury to the nasal architecture and mucosa, and perhaps greater clinical utility in managing respiratory distress in premature infants in middle-income countries.

We believe our experience calls for a large multicenter randomized controlled trial comparing the efficacy, safety, and cost-benefit of HHHFNC to NCPAP in China in the future.

## 6. Conclusion

Our study shows that HHHFNC is an effective and well-tolerated strategy that could be as effective as NCPAP as the primary treatment of mild to moderate RDS in preterm infants between 28^+0^ and 34^+6^ weeks' GA. Multicenter randomized clinical trials should be conducted to verify our findings concerning the use of HHHFNC as primary respiratory support for preterm infants with RDS in low- and middle-income countries in the future.

## Figures and Tables

**Table 1:** Demographic characteristics of the study population.

Characteristic	HHHFNC (*n* = 65)	NCPAP (*n* = 63)
Gestational age, week, mean (SD)	31.9 (1.7)	31.9 (1.8)
<32 weeks, *n* (%)	20 (30.8)	22 (34.9)
Birth weight, mean ± SD, g	1754 (299)	1790 (373)
<1500 g, *n* (%)	16 (24.6)	16 (25.4)
Small for gestational age, number (%)	3 (4.6)	4 (6.3)
Female, number (%)	35 (53.8)	30 (47.6)
Multiple birth, number (%)	14 (21.5)	16 (25.4)
Antenatal steroids, number (%)	26 (40)	23 (36.5)
Cesarean delivery, number (%)	35 (53.8)	31 (49.2)
Neonatal resuscitation, number (%)	38 (58.5)	35 (55.6)
Apgar score at 5 min, median (IQR)	9 (8-9)	9 (8-9)
Prestudy surfactant, *n* (%)	20 (30.8)	22 (34.9)
Prestudy caffeine, *n* (%)	24 (36.9)	25 (39.7)
pH before enrollment, mean (SD)	7.19 (0.06)	7.19 (0.06)
PCO_2_ before enrollment, mean (SD), mmHg	55.3 (3.3)	55.5 (3.5)
FIO_2_ before enrollment, median (IQR)	0.23 (0.21–0.30)	0.25 (0.21–0.30)

FIO_2_, fraction of inspired oxygen; HHHFNC, heated, humidified high-flow nasal cannula; IQR, interquartile range; NCPAP, nasal continuous positive airway pressure; PCO_2_, partial pressure of carbon dioxide.

*P* > 0.05 for all comparisons.

**Table 2:** Primary outcome results.

Outcome	HHHFNC (*n* = 65)	NCPAP (*n* = 63)	95% CI of risk difference	*P*value^a^
Mechanical ventilation within 7 days, number (%)	13 (20)	11 (17.5)	0.5 to 2.9	0.71
Gestational age^b^				
28^+0^ to 32^+6^	9 (23.7)	8 (21.1)	0.4 to 3.4	0.78
33^+0^ to 34^+6^	4 (14.8)	3 (12)	0.3 to 6.4	0.77
Age at the start of mechanical ventilation, median (IQR), h	35.2 (6–90)	22.3 (4–80)	−8.35 to 34.12	0.21
Duration of mechanical ventilation, median (IQR), d	3.3 (1 to 5)	3.5 (2 to 5)	−1.32 to 0.84	0.87

HHHFNC, heated, humidified high-flow nasal cannula; IQR, interquartile range; NCPAP, nasal continuous positive airway pressure. ^a^Dichotomous outcomes were compared by *χ*^2^ test; continuous outcomes were compared by Wilcoxon 2-sample test. ^b^Gestational age is presented as weeks^+days^.

**Table 3:** Respiratory support outcomes among infants assigned to HHHFNC compared with NCPAP.

Outcome	HHHFNC (*n* = 65)	NCPAP (*n* = 63)	95% CI of risk difference	*P* value
Duration received, median (IQR), d				
Respiratory support	5.6 (3 to 15)	5.1 (2 to 14)	−0.32 to 1.33	0.72
Noninvasive respiratory support	5.2 (3 to 13)	4.8 (2 to 13)	−0.25 to 1.06	0.31
Oxygen supplementation	0.4 (0 to 3)	0.3 (0 to 3)	−0.19 to 0.38	0.26
Caffeine treatment	3.2 (0 to 24)	2.0 (0 to 22)	−0.23 to 2.51	0.68
Surfactant, number (%)	25 (38.5)	27 (42.9)	0.41 to 1.69	0.61
Air leaks	2 (3.1)	1 (1.6)	0.17 to 22.3	0.58
BPD	6 (9.2)	6 (9.5)	0.29 to 3.2	0.96
Age at discharge, d	30.5 (14 to 55)	30.6 (16 to 49)	−4.08 to 3.86	0.96

BPD, bronchopulmonary dysplasia; HHHFNC, heated, humidified high-flow nasal cannula; IQR, interquartile range.

**Table 4:** Occurrence rates for secondary outcomes in the HHHFNC compared with the NCPAP study group.

Outcome	HHHFNC (*n* = 65)	NCPAP (*n* = 63)	95% CI of risk difference	*P* value
Adverse event, number (%)				
Confirmed sepsis	5 (7.7)	7 (11.1)	0.20 to 2.22	0.51
IVH	3 (4.6)	2 (3.2)	0.24 to 9.14	0.67
PDA	4 (6.2)	4 (6.3)	0.23 to 4.05	0.96
ROP	3 (4.6)	2 (3.2)	0.24 to 9.14	0.67
nasal trauma	14 (21.5)	27 (42.9)	0.17 to 0.79	0.01
Death	1 (1.5)	1 (1.6)	0.06 to 16.08	0.99
Abdominal distention	7 (10.8)	8 (12.7)	0.28 to 2.44	0.73
Days to full oral feedings, median (IQR), d	11.1 (5–20)	11.5 (5–24)	−2.30 to 1.50	0.68
Exclusive breastfeeding at discharge, number (%)	12 (18.5)	10 (15.9)	0.48 to 3.02	0.70
Weight at discharge, median (IQR), g	2150 (2000 to 2450)	2176 (2050 to 2550)	−73.5 to 22.5	0.30
